# Interaction between Age and Primary Site on Survival Outcomes in Primary GI Melanoma over the Past Decade

**DOI:** 10.3390/medsci11020032

**Published:** 2023-04-28

**Authors:** Ayrton Bangolo, Pierre Fwelo, Sowmya Sagireddy, Harin Shah, Chinmay Trivedi, John Bukasa-Kakamba, Rutvij Patel, Luke Bharane, Manraj K. Randhawa, Vignesh K. Nagesh, Shraboni Dey, Hannah Terefe, Gagan Kaur, Nicholas Dinko, Fatma Lina Emiroglu, Ahmed Mohamed, Mark A. Fallorina, David Kosoy, Danish Waqar, Ankita Shenoy, Kareem Ahmed, Anvit Nanavati, Amritpal Singh, Anthony Willie, Diego M. C. Gonzalez, Deblina Mukherjee, Jayadev Sajja, Tracy Proverbs-Singh, Sameh Elias, Simcha Weissman

**Affiliations:** 1 Department of Internal Medicine, Hackensack Meridian Health/Palisades Medical Center, North Bergen, NJ 07047, USA; 2 Department of Epidemiology, Human Genetics, and Environmental Sciences, UTHealth School of Public Health, Houston, TX 77030, USA; 3 Department of Endocrinology, Kinshasa University Clinics, Kinshasa 190, Democratic Republic of the Congo; 4 Department of Internal Medicine, University of Washington, Seattle, WA 98195, USA; 5 Department of Gastrointestinal Malignancies, Hackensack Meridian Health/John Theurer Cancer Center, North Bergen, NJ 07047, USA

**Keywords:** primary GI melanoma, SEER database, prognostic factors, cancer, mortality

## Abstract

Background: Primary malignant melanomas of the Gastrointestinal mucosa are uncommon. Most cases of gastrointestinal (GI) melanomas are secondary, arising from metastasis at distant sites. The purpose of this study is to assess to what extent the interaction between independent prognostic factors (age and tumor site) of primary GI melanoma influence survival. Furthermore, we also aimed to investigate the clinical characteristics, survival outcomes, and independent prognostic factors of patients with primary GI melanoma in the past decade. Methods: A total of 399 patients diagnosed with primary GI melanoma, between 2008 and 2017, were enrolled in our study by retrieving data from the Surveillance, Epidemiology, and End Results (SEER) database. We analyzed demographics, clinical characteristics, and overall mortality (OM) as well as cancer-specific mortality (CSM) of primary GI melanoma. Variables with a *p* value < 0.1 in the univariate Cox regression were incorporated into the multivariate Cox model (model 1) to determine the independent prognostic factors, with a hazard ratio (HR) of greater than 1 representing adverse prognostic factors. Furthermore, we analyzed the effect of the interaction between age and primary location on mortality (model 2). Results: Multivariate cox proportional hazard regression analyses revealed higher OM in age group 80+ (HR = 5.653, 95% CI 2.212–14.445, *p* = 0), stomach location of the tumor (HR = 2.821, 95% CI 1.265–6.292, *p* = 0.011), regional lymph node involvement only (HR = 1.664, 95% CI 1.051–2.635, *p* < 0.05), regional involvement by both direct extension and lymph node involvement (HR = 1.755, 95% CI 1.047–2.943, *p* < 0.05) and distant metastases (HR = 4.491, 95% CI 3.115–6.476, *p* = 0), whereas the lowest OM was observed in patients with small intestine melanoma (HR = 0.383, 95% CI 0.173–0.846, *p* < 0.05). Multivariate cox proportional hazard regression analyses of CSM also revealed higher mortality of the same groups and lower CSM in small intestine and colon melanoma excluding the rectum. For model 2, considering the interaction between age and primary site on mortality, higher OM was found in age group 80+, followed by age group 40–59 then age group 60–79, regional lymph node involvement only, regional involvement by both direct extension and lymph node involvement and distant metastases. The small intestine had a lower OM. The rectum as primary location and the age range 40–59 interacted to lower the OM (HR = 0.14, 95% CI 0.02–0.89, *p* = 0.038). Age and primary gastric location did not interact to affect the OM. For the CSM, taking into account the interaction between age and the primary location, higher mortality was found in the same groups and the colon location. The primary colon location also interacted with the age group 40–59 to increase the CSM (HR = 1.38 × 10^9^, 95% CI 7.80 × 10^7^–2.45 × 10^10^, *p* = 0). Conclusions: In this United States population-based retrospective cohort study using the SEER database, we found that only the age range 40–59 interacted with the rectum and colon to lower and increase mortality respectively. Primary gastric location, which was the single most important location to affect mortality, did not interact with any age range to influence mortality. With those results, we hope to shed some light on this rare pathology with a very dismal prognosis.

## 1. Introduction

Melanoma accounts for approximately 6% of all primary cancers in the United States. Most melanoma cases are cutaneous in origin [1]. When present in the gastrointestinal (GI) tract, melanomas are thought to originate from a primary cutaneous origin [2].

To date, only a few cases of primary GI melanomas have been reported in the literature and are believed to arise from ectopic melanocytes [3,4,5,6,7,8,9].

Melanoma of the GI tract is a rare occurrence that can carry a poor prognosis. The primary site of the melanoma is usually the skin and metastases within the GI tract commonly occur in the liver, small intestine, colon, and stomach in decreasing order of incidence [6]. The incidence of primary GI melanoma is low in that it only represents 2–15% of all cases of GI melanomas [10].

Based on the prior literature, there have been very few studies that have addressed the survival outcome of patients with primary GI melanoma. The study by Zheng et al., which enrolled patients from 1975 to 2016, is one of the largest studies addressing the epidemiological characteristics of primary GI melanomas [11]. The study demonstrated that while the incidence of primary GI melanomas has been increasing since 1975, they are still rare and are detected at advanced stages [11]. The study addressed independent prognostic factors. However, to the best of our knowledge, there are currently no studies in the literature assessing the interaction between independent prognostic factors and how they influence mortality, making our study the first of its kind.

We used a nationally representative database to evaluate the independent prognostic factors amongst patients with primary GI melanomas. Furthermore, we evaluated the interaction between them and the influence of such interactions on mortality. While there has been a stable number of yearly new diagnoses of primary GI melanoma over the past decade, we believe there has been a higher mortality in patients with primary gastric location and older patients. However, we believe that these two independent prognostic factors do not interact to affect mortality. We hope that this study will pave the path for future larger cohort studies in regard to evaluating the prognostic factors associated with primary GI melanomas and factors impacting survival outcomes in these patients, focusing on their interaction.

## 2. Materials and Methods

### 2.1. Study Design

A population-based retrospective cohort study of patients with primary GI melanoma was conducted using the SEER research plus data, 18 registries, November 2020 submission database (http://www.seer.cancer.gov, accessed on 4 April 2020). The SEER Program is one of the largest and most authoritative sources of the cancer-related data in the United States, and is sponsored by the United States National Cancer Institute (US NCI). The SEER 18 database collects cancer incidence, patients’ clinicopathological features, and survival data from 18 population-based cancer registries and covers nearly 28% of the US population [12].

### 2.2. Data Selection

#### 2.2.1. Inclusion Criteria

All patients with primary GI melanoma diagnosed from 2007 to 2018 were selected in our cohort based on (1) primary site (c15.0 to c21.8 and c26 to c26.9) and (2) histological type (8720 to 8790) [11]. The above-mentioned ICD-9 and/or ICD-10 codes were used to extract data regarding these patients from the SEER database.

#### 2.2.2. Exclusion Criteria

We excluded patients with an unknown age at diagnosis, race, or stage of GI melanoma.

### 2.3. Study Variables

#### 2.3.1. Main Exposure

Tumor sites, age and the interaction between them were the main predictors of mortality in this study. Tumor sites were classified into stomach, anus, colon, small intestine, esophagus, rectum, and others. Age was classified as <30 years old, 40–59 years old, 60–79 years old and 80+ years old.

#### 2.3.2. Outcomes

Overall mortality: Patients who died of any causes at end of the study were categorized as “yes”, and those who did not were categorized as “no”. The total number of deaths from any cause among patients with primary GI melanoma yearly, between 2008 and 2017, was evaluated.

Cancer-specific mortality: Patients who died of primary GI melanoma at the end of the study were categorized as “yes”, and those who died of other causes were classified as “no”. Total number of deaths, with primary GI melanoma as the underlying cause of death, occurring in patients with primary GI melanoma yearly, between 2008 and 2017, was evaluated.

#### 2.3.3. Survival Months

For overall mortality, survival time was calculated from the date of diagnosis to the date of death, or the date of last follow-up (31 December 2017) as reported in the SEER registry. For the cancer-specific mortality, survival time was calculated from the date of diagnosis to the date of GI-melanoma-related death, or the date of last follow-up as recorded in the SEER registry.

#### 2.3.4. Sociodemographic and Tumor Characteristics

Variables such as age at diagnosis, gender, race (White, Black, and others: American Indian/Alaskan Native and Asian/Pacific Islander combined), origin (Non-Hispanic and Hispanic), year of diagnosis, primary site of tumor, histological type, stage at diagnosis (localized, regional, and distant), geographic residential area, yearly income, marital status, year of diagnosis, surgery, radiation, and chemotherapy were extracted. The yearly income provided correlated to the following social classes: Poverty class (<USD 35,000), lower class (USD 35,000–44,999), working class (USD 45,000–54,999), middle class (USD 55,000–64,999), upper-middle class (USD 65,000–74,999) and upper class (USD 75,000+).

Histologic characteristics were categorized as melanoma not otherwise specified (NOS), nodular melanoma, spindle cell melanoma NOS, mucosal lentiginous melanoma, and others. “Malignant melanoma, NOS” indicates no tumor subtype in patient records.

### 2.4. Statistical Analysis

A Cox proportional hazard regression model is based on the assumption that hazard rates are proportional over time. Variables with a *p* value < 0.1 in the univariate Cox regression model were incorporated into the multivariate Cox proportional analysis to determine the prognostic factors associated with OM and CSM (model 1). In the second model, we adjusted for the variables in model 1 plus the interaction between age at diagnosis and tumor site with a hazard ratio (HR) > 1 representing adverse prognostic factors. All tests were two-sided, with a confidence interval set as 95% and a *p* value < 0.05 deemed statistically significant. All statistical tests were performed using Software STATA 17.

## 3. Results

A total of 399 patients with primary GI melanoma were included in our study. The general demographic and clinicopathological characteristics of this cohort are summarized in Table 1. The male gender (56.14%), age range 60–79 (52.88%), non-Hispanic whites (70.18%), counties in metropolitan areas of 1 million people (63.41%), and yearly income USD 75,000+ (30.33) were the most represented groups in our cohort. The most encountered primary site was the anus (51.88%), which represented more than half the cases. Malignant melanoma NOS (86.97%) was the most common histologic type, and spindle cell melanoma NOS (1.25%) was the least common. The majority of diagnoses were made at the advanced disease stage, with distant metastases (41.35%). Married patients constituted most of the study (53.13%), followed by widowed patients (20.05%). Most patients did not undergo surgical resection (84.71%) or receive chemotherapy (79.45%). There was a steady number of new cases from 2008 to 2017 with an average of 40 new cases per year.

A crude analysis of factors associated with all-cause mortality and primary gastrointestinal melanoma related mortality among US patients between 2008 and 2017 is demonstrated in Table 2. Age 80+ (HR = 4.042, 95% CI 1.732–9.433, *p* = 0.001), followed by age 60–79 (HR = 2.296, 95% CI 1.01–5.216, *p* = 0.047); gastric primary location (HR = 3.261, 95% CI 1.658–6.417, *p* = 0.001); primary GI melanoma with distant metastases (HR = 2.967, 95% CI 2.22–3.965, *p* = 0); nonmetropolitan counties not adjacent to a metropolitan area (HR = 2.211, 95% CI 1.253–3.9, *p* = 0.006); and chemotherapy (HR = 1.417, 95% CI 1.078–1.863, *p* = 0.012) have the highest overall mortality. The highest cancer-specific mortality was observed in age 80+ (HR = 3.343, 95% CI 1.424–7.844, *p* = 0.006); primary gastric location (HR = 3.108, 95% CI 1.518–6.367, *p* = 0.002), followed by colon (HR = 0.221, 95% CI 0.55–0.895, *p* = 0.055); advanced disease with distant metastasis (HR = 3.309, 95% CI 2.421–4.522, *p* = 0), followed by regional lymph node involvement (HR = 1.632, 95% CI 1.058–2.516, *p* = 0.027); nonmetropolitan counties not adjacent to a metropolitan area (HR = 2.376, 95% CI 1.344–4.2, *p* = 0.003); and chemotherapy (HR = 1.45, 95% CI 1.09–1.928, *p* = 0.01).

Multivariate cox proportional hazard regression analyses of factors affecting all-cause mortality and primary gastrointestinal melanoma related mortality among US patients between 2008 and 2017, not considering the interaction between age and primary location (model 1), are demonstrated in Table 3. Higher overall mortality was observed in age 80+ (HR = 5.653, 95% CI 2.212–14.445, *p* = 0), followed by age 60–79 (HR = 3.062, 95% CI 1.26–7.442, *p* = 0.014); gastric location of melanoma (HR = 2.821, 95% CI 1.265–6.292, *p* = 0.011); advanced disease with distant metastasis (HR = 4.491, 95% CI 3.115–6.476, *p* = 0), followed by regional involvement by both direct extension and lymph node involvement (HR = 1.755, 95% CI 1.047–2.943, *p* = 0.033). Age 80+ (HR = 4.654, 95% CI 1.79–12.104, *p* = 0.002), followed by age 60–79 (HR = 2.815, 95% CI 1.149–6.898, *p* = 0.024); primary gastric location (HR = 3.05, 95% CI 1.307–7.119, *p* = 0.01); advanced disease with distant metastases (HR = 5.091, 95% CI 3.424–7.568, *p* = 0), followed by regional involvement by both direct extension and lymph node involvement (HR = 2.023, 95% CI 1.177–3.479, *p* = 0.011) have the highest cancer-specific mortality. In model 2, taking into account the interaction between the primary location and the age at diagnosis, higher OM was found in age group 80+, followed by age group 40–59 then age group 60–79, regional lymph node involvement only, regional involvement by both direct extension and lymph node involvement and distant metastases. The small intestine had a lower OM. The rectum as primary location and the age range 40–59 interacted to lower the OM (HR = 0.14, 95% CI 0.02–0.89, *p* = 0.038). Age and primary gastric location did not interact to affect the OM. For the CSM, taking into account the interaction between age and the primary location, higher mortality was found in the same groups and the colon location. The primary colon location also interacted with the age group 40–59 to increase the CSM (HR = 1.38 × 10^9^, 95% CI 7.80 × 10^7^–2.45 × 10^10^, *p* = 0).

## 4. Discussion

In this large SEER data-based retrospective cohort study, we found that advanced age, primary gastric location, and advanced disease are independent prognostic factors associated with higher OM and CSM, whereas small intestine had a lower OM and CSM. Primary colon location had a lower CSM. When accounting for the interaction between age and primary location, gastric location did not interact with any age group. The rectum as primary location interacted with the age range 40–59 to lower OM while the colon interacted with the same age range to increase the CSM.

Primary non cutaneous melanomas are rare. Only 20% of them arise from mucosal sites and, of these, 25% are found in the GI tract [13]. Primary GI melanomas have been associated with poor prognosis and aggressive behavior [11]. However, given its rare occurrence, only a few adequately powered studies have addressed the epidemiology and prognosis factors [11,14,15].

Primary gastric location was associated with the highest overall mortality and cancer-specific mortality in our cohort. Similar results were found in the Zheng series [11]. However, overall mortality of gastric location in the multivariate analysis was slightly higher (HR = 2.821, 95% CI 1.265–6.292, *p* = 0.011) in our cohort compared to the Zheng study (HR = 2.47, 95% CI 1.73–3.50, *p* = 0.000) [11], which can be explained by a smaller sample size and a shorter period of study in our cohort. However, when accounting for the interaction between age and location, the primary gastric location did not affect mortality in our cohort, an aspect that was not uncovered by the Zheng study. Small intestine location has the lowest overall mortality in our cohort compared to other locations. This finding differs from the Zheng series in which the anal and colonic melanoma had better overall survival than other GI melanoma subtypes [11]. However, when accounting for the interaction between the age and the location, colonic melanoma had the worst CSM among patients in the age group 40–59, another aspect that was not uncovered in previous studies. A somehow similar trend was seen in a study of patients with stage III colon carcinoma; where younger patients (50–69 years old) were more likely to die from colon cancer related death than their older counterparts (70+ years old) (81% VS 62%) [16]. Furthermore, rectal melanoma interacted with the same age group to lower OM. This can be explained by the fact that most patients with rectal cancer are more likely to present with GI bleeding [17] as compared to more proximal GI cancers which may prompt them to undergo initial screening colonoscopy, thus rectal melanoma may be diagnosed at a localized stage in this age group and earlier intervention may be taken.

Our study revealed a male gender predominance, which differed from the Zheng series and the Al-Haseni series, which found a higher proportion of females [11,14]. Most patients were diagnosed between the ages of 60–79 (52.88%); a similar trend was observed in the Zheng series (48.9%). The rectum and the anus were the most common primary sites, consistent with the literature [11,14,15]. This can be explained by the fact that cancer at these locations (rectum and anus) is more likely to present with lower GI bleed and prompting the need for an endoscopic evaluation [17]. Populated areas and higher income were associated with higher diagnosis in our cohort. This could be explained by nonspecific symptoms of primary GI melanoma and the need for advanced and costly diagnostic imaging to make the diagnosis of primary GI melanoma [18,19]. Patients living in populated metropolitan areas have more access to advanced imaging and healthcare services, and patients with higher income are more likely to afford the diagnostic means.

Several epidemiologic cancer studies have found marital status to be an independent prognostic factor. Married patients were found to have a lower overall and cancer-specific mortality, compared to their non-married counterparts [20,21,22,23,24,25,26,27,28,29]. This was mainly thought to be due to better social support among married patients. However, in our cohort, marital status did not significantly impact the overall mortality or cancer-specific mortality. There was no difference in mortality between married and non-married patients. This can be explained by the fact that the primary GI melanoma is often diagnosed at advanced stages and social support might not really affect the outcome.

Age 80+ and primary GI melanoma with distant metastases are also associated with higher overall mortality in the univariate analysis. Similar findings are seen in the literature [11]. Interestingly, as noted in our univariate analysis, the same variables i.e., age 80+ and advanced disease with distant metastasis, were also associated with higher overall and cancer-specific mortality in our multivariate regression analysis. However, when accounting for the tumor location, which was also an important independent factor to affect mortality, age 80+ did not interact with tumor site to influence mortality. These are findings not previously addressed in the literature. Elderly patients usually have immunosenescence and/or other associated comorbidities which decreases their ability to fight off the cancer cells [1,2,3]. Furthermore, primary GI melanomas with distant metastasis are diagnosed very late and not many novel therapies are available currently to target such an advanced cancer, and hence they may be associated with poor prognosis, as evidenced in our study. Intuitively, it makes sense to assume that patients who have early detection of their cancer could possibly have a better overall as well as a cancer-specific survival outcome associated with a good prognosis. However, to conclusively state that, further larger cohort studies are warranted. Our study sets the stage for future larger studies on the subject to evaluate whether more stringent monitoring could possibly lead to detection of these primary GI melanomas at an early stage and how early detection affects overall as well as the cancer-specific mortality.

Additionally, nonmetropolitan counties not adjacent to a metropolitan area and chemotherapy also have higher overall mortality, as noted in our univariate analysis. The patients residing in non-metropolitan counties may not have access to higher tertiary care centers and advanced healthcare facilities in close vicinity, which significantly decreases the ability to maintain regular follow-up visits. Other financial factors as well as sub-optimal healthcare delivery could also be playing a role. However, in the multivariate regression, chemotherapy and residential areas did not yield a higher overall or cancer-specific mortality. Furthermore, surgical resection of primary tumors was associated with lower mortality in the Zheng series [11]. However, in our cohort, there was no statistically significant difference between surgical resection and non-surgical resection. This difference can be explained by the fact that our study had a smaller sample size.

Certain limitations must be considered when interpreting the results of this study. Our study was mainly carried out on primary GI melanoma, which makes it difficult to generalize our results to metastatic GI melanomas. Information gathered on patients who underwent radiotherapy was not complete. Furthermore, the publicly available SEER database does not provide information on comorbidities. However, this study has the merit of collecting data from the largest cancer database in the USA. Furthermore, we were also able to enroll an adequate sample size despite the rarity of the pathology. Our study also has the merit of being the first of its kind addressing such an important clinical question.

## 5. Conclusions

There is paucity of prior research on primary GI melanomas, owing to the rarity of the condition. They are associated with aggressive behavior and poor prognosis. Our cohort focused on a 10-year period and demonstrated that although advanced age (80+) and primary gastric location are important independent factors of prognosis, they did not interact to influence mortality. Furthermore, middle-aged patients (40–59) interacted with the colon and the rectum to affect mortality. We hope that the results of this study will shed light on this important interaction between middle age and primary location regarding mortality amongst primary GI melanoma patients and perhaps inspire larger prospective studies on this subject.

## Figures and Tables

**Table 1:** Demographic and clinicopathologic characteristics of US patients diagnosed with primary GI melanoma between 2008 and 2017.

Characteristics		
	***N* =**	**%**
**Total**	399	100
**Gender**		
Male	224	56.14
Female	175	43.86
**Age at diagnosis, years old**		
0–39	14	3.51
40–59	100	25.06
60–79	211	52.88
80+	74	18.55
**Race**		
Non-Hispanic white	280	70.18
Non-Hispanic black	19	4.76
Hispanic	64	16.04
Other	36	9.02
**Cancer Site**		
Anus	207	51.88
Colon	10	2.51
Esophagus	17	4.26
Rectum	130	32.58
Small intestine	17	4.26
Stomach	12	3.01
Other	6	1.50
**Histologic Subtype**		
Malignant melanoma not otherwise specified	347	86.97
Nodular melanoma	34	8.52
Spindle cell melanoma not otherwise specified	5	1.25
Mucosal lentiginous melanoma	6	1.50
Other	7	1.75
**Tumor stage**		
Localized	127	31.83
Regional by direct extension only	25	6.27
Regional lymph nodes involved only	49	12.28
Regional by both direct extension and lymph node involvement	33	8.27
Distant	165	41.35
**Living area**		
Counties in metropolitan areas of 1 million persons	253	63.41
Counties in metropolitan areas of 250,000 to 1 million persons	72	18.05
Counties in metropolitan areas of 250,000 persons	32	8.02
Nonmetropolitan counties adjacent to a metropolitan area	28	7.02
Nonmetropolitan counties not adjacent to a metropolitan area	14	3.51
**Income per year**		
<USD 35,000	6	1.50
USD 35,000–44,999	28	7.02
USD 45,000–54,999	65	16.29
USD 55,000–64,999	87	21.80
USD 65,000–74,999	92	23.06
USD 75,000+	121	30.33
**Marital Status**		
Married	212	53.13
Single	58	14.54
Divorced/separated	29	7.27
Widowed	80	20.05
Unknown	20	5.01
		
**Surgery and Radiation**		
Yes	61	15.29
No	338	84.71
		
**Chemotherapy**		
Yes	82	20.55
No	317	79.45
		
**Year of diagnosis**		
2008	34	8.52
2009	46	11.53
2010	34	8.52
2011	30	7.52
2012	38	9.52
2013	32	8.02
2014	50	12.53
2015	50	12.53
2016	39	9.77
2017	46	11.53

**Table 2:** Crude analysis of factors associated with all-cause mortality and primary gastrointestinal melanoma related mortality among US patients between 2008 and 2017.

Characteristics	Overall Mortality. Crude Proportional Hazard Ratio (95% Confidence Interval)	Primary GI Melanoma Mortality. Crude Proportional Hazard Ratio (95% Confidence Interval)
**Age, years old**		
0–39	1.00 (reference)	1.00 (reference)
40–59	1.983 (0.857–4.588)	1.822 (0.786–4.223)
60–79	2.296 (1.01–5.216) **	2 (0.879–4.553)
80+	4.042 (1.732–9.433) ***	3.343 (1.424–7.844) ***
**Race**		
Non-Hispanic white	1.00 (reference)	1.00 (reference)
Non-Hispanic black	1.006 (0.585–1.731)	0.86 (0.468–1.582)
Hispanic	1.017 (0.738–1.403)	0.961 (0.682–1.354)
Other	0.814 (0.524–1.266)	0.807 (0.509–1.282)
**Cancer Site**		
Anus	1.00 (reference)	1.00 (reference)
Colon	0.5 (0.205–1.22)	0.221 (0.55–0.895) **
Esophagus	1.642 (0.964–2.795)	1.662 (0.958–2.885)
Stomach	3.261 (1.658–6.417) ***	3.108 (1.518–6.367) ***
Rectum	1.128 (0.867–1.467)	1.118 (0.849–1.472)
Small intestine	0.654 (0.333–1.284)	0.639 (0.313–1.307)
Other	1.462 (0.539–3.966)	1.215 (0.385–3.834)
**Histologic Subtype**		
Malignant Melanoma, not otherwise specified	1.00 (reference)	1.00 (reference)
Nodular melanoma	0.986 (0.653–1.489)	0.966 (0.623–1.498)
Spindle cell melanoma, not otherwise specified	0.379 (0.094–1.525)	0.428 (0.106–1.723)
Mucosal lentiginous melanoma	0.508 (0.163–1.59)	0.574 (0.183–1.795)
Other	0.375 (0.12–1.171)	0.423 (0.135–1.323)
**Tumor stage**		
Localized	1.00 (reference)	1.00 (reference)
Regional by direct extension only	0.968 (0.547–1.715)	1.086 (0.596–1.977)
Regional lymph nodes involved only	1.482 (0.986–2.225)	1.632 (1.058–2.516) **
Regional by both direct extension and lymph node involvement	1.374 (0.873–2.164)	1.587 (0.989–2.546)
Distant	2.967 (2.22–3.965) ***	3.309 (2.421–4.522) ***
**Living area**		
Counties in metropolitan areas of 1 million persons	1.00 (reference)	1.00 (reference)
Counties in metropolitan areas of 250,000 to 1 million persons	1.014 (0.742–1.386)	0.954 (0.683–1.333)
Counties in metropolitan areas of 250,000 persons	0.866 (0.539–1.39)	0.895 (0.549–1.457)
Nonmetropolitan counties adjacent to a metropolitan area	1.091 (0.693–1.718)	0.964 (0.584–1.592)
Nonmetropolitan counties not adjacent to a metropolitan area	2.211 (1.253–3.9) ***	2.376 (1.344–4.2) ***
**Income per year**		
<USD 35,000	1.00 (reference)	1.00 (reference)
USD 35,000–44,999	1.005 (0.38–2.656)	1.206 (0.414–3.515)
USD 45,000–54,999	0.725 (0.289–1.824)	0.842 (0.302–2.345)
USD 55,000–64,999	0.634 (0.254–1.581)	0.709 (0.256–1.962)
USD 65,000–74,999	0.617 (0.248–1.535)	0. 697 (0.252–1.925)
USD 75,000+	0.555 (0.224–1.371)	0.643 (0.235–1.763)
**Marital Status**		
Married	1.00 (reference)	1.00 (reference)
Single	0.994 (0.691–1.43)	1.029 (0.707–1.497)
Divorced/separated	1.011 (0.634–1.612)	0.78 (0.45–1.352)
Widowed	1.074 (0.788–1.465)	1.071 (0.773–1.483)
**Surgery and Radiation**		
No	1.00 (reference)	1.00 (reference)
Yes	0.796 (0.574–1.102)	0.78 (0.552–1.102)
**Chemotherapy**		
No	1.00 (reference)	1.00 (reference)
Yes	1.417 (1.078–1.863) **	1.45 (1.09–1.928) **
**Year of diagnosis**		
2008	1.00 (reference)	1.00 (reference)
2009	1.308 (0.804–2.127)	1.416 (0.843–2.378)
2010	1.289 (0.76–2.189)	1.347 (0.764–2.375)
2011	1.175 (0.672–2.051)	1.214 (0.665–2.213)
2012	1.006 (0.589–1.719)	1.093 (0.619–1.929)
2013	1.101 (0.645–1.88)	1.238 (0.705–2.174)
2014	1.018 (0.615–1.685)	1.082 (0.632–1.854)
2015	0.856 (0.509–1.439)	0.828 (0.471–1.456)
2016	0.888 (0.5–1.579)	1.017 (0.56–1.846)
2017	0.764 (0.418–1.399)	0.722 (0.374–1.395)

*** *p* < 0.01, ** *p* < 0.05.

**Table 3:** Multivariate cox proportional hazard regression analyses of factors affecting all-cause mortality and primary gastrointestinal melanoma related mortality among US patients between 2008 and 2017, with model 1 not considering the interaction between age and tumor site and model 2 taking into account the interaction between age and tumor site.

Characteristics	Overall Mortality. Adjusted Proportional Hazard Ratio (95% Confidence Interval) Model 1	Primary GI Melanoma Mortality. Adjusted Proportional Hazard Ratio (95% Confidence Interval) Model 1	Overall Mortality. Adjusted Proportional Hazard Ratio (95% Confidence Interval) Model 2	Primary GI Melanoma Mortality. Adjusted Proportional Hazard Ratio (95% Confidence Interval) Model 2
				
**Gender**				
Female	1.345 (0.99–1.828)	1.164 (0.841–1.609)	1.00 (reference)	1.00 (reference)
Male	1.00 (reference)	1.00 (reference)	1.35 (0.99–1.828)	1.20 (0.861–1.86)
				
**Age, years old**				
0–39	1.00 (reference)	1.00 (reference)	1.00 (reference)	1.00 (reference)
40–59	2.612 (1.05–6.495) **	2.495 (0.996–6.251)	5.57 (1.58–19.66) ***	5.1 (1.44–18.28) **
60–79	3.062 (1.26–7.442) **	2.815 (1.149–6.898) **	4.81 (1.38–16.75) **	4.46 (1.28–15.57) **
80+	5.653 (2.212–14.445) ***	4.654 (1.79–12.104) ***	8.99 (2.49–32.36) ***	6.76654 (1.85–24.73) ***
				
**Race**				
Non-Hispanic white	1.00 (reference)	1.00 (reference)	1.00 (reference)	1.00 (reference)
Non-Hispanic black	0.79 (0.418–1.49)	0.625 (0.306–1.275)	0.98 (0.5–1.93)	0.83 (0.39–1.75)
Hispanic	0.988 (0.677–1.442)	0.949 (0.635–1.418)	1.11 (0.75–1.64)	1.08 (0.71–1.63)
Other	0.926 (0.567–1.514)	0.912 (0.546–1.524)	0.93 (0.56–1.52)	0.9 (0.54–1.51)
				
**Cancer Site**				
Anus	1.00 (reference)	1.00 (reference)	1.00 (reference)	1.00 (reference)
Colon	0.489 (0.187–1.283)	0.23 (0.054–0.979) **	0.24 (0.3–1.97)	2.43 × 10^−10^ (3.21 × 10^−11^–1.84 × 10^−9^) ***
Esophagus	1.376 (0.736–2.57)	1.315 (0.686–2.521)	1.85 (0.61–5.61)	2.19 (0.71–6.78)
Stomach	2.821 (1.265–6.292) **	3.05 (1.307–7.119) ***	1.77 (0.36–8.68)	2.48 (0.49–12.5)
Rectum	1.039 (0.768–1.405)	1.012 (0.738–1.388)	3.54 (0.62–20.08)	3.14 (0.55–17.85)
Small intestine	0.383 (0.173–0.846) **	0.375 (0.163–0.864) **	0.38 (0.16–0.89) **	0.36 (0.15–0.90)
Other	0.81 (0.267–2.452)	0.627 (0.179–2.2)	0.74 (0.15–3.59)	0.35 (0.04–2.88)
				
**Interaction between age (years old) and tumor site**				
0–39 and tumor sites	NA	NA	1.00 (reference)	1.00 (reference)
40–59 and colon	NA	NA	1.32 (0.74–23.50)	1.38 × 10^9^ (7.80 × 10^7^–2.45 × 10^10^) ***
40–59 and esophagus	NA	NA	0.45 (0.08–2.33)	0.29 (0.49–1.77)
40–59 and stomach	NA	NA	1.13 (0.18–6.97)	0.77 (0.12–5.12)
40–59 and rectum	NA	NA	0.14 (0.02–0.89) **	0.16 (0.02–1.03)
40–59 and small intestine	NA	NA	1.81 (0.18–17.69)	1.94 (0.19–19.74)
40–59 and others	NA	NA	0.99 (0.11–9.28)	2.25 (0.16–31.72)
60–79 and colon	NA	NA	4.33 (0.38–48.89)	1.86 × 10^9^
60–79 and esophagus	NA	NA	0.79 (0.17–3.68)	0.62 (0.13–2.93)
60–79 and stomach	NA	NA	5.01 (0.56–44.44)	3.97 (0.44–36.18)
60–79 and rectum	NA	NA	0.33 (0.05–1.99)	0.35 (0.06–2.11)
60–79 and small intestine	NA	NA	1	1
60–79 and other	NA	NA	1	1
80+ and colon	NA	NA	1	1
80+ and esophagus	NA	NA	1	1
80+ and stomach	NA	NA	1	1
80+ and rectum	NA	NA	0.91 (0.08–3.03)	0.61 (0.098–3.85)
80+ and small intestine	NA	NA	1	1
80+ other	NA	NA	1	1
				
**Histologic Subtype**				
Malignant melanoma, not otherwise specified	1.00 (reference)	1.00 (reference)	1.00 (reference)	1.00 (reference)
Nodular melanoma	1.011 (0.627–1.632)	0.94 (0.564–1.569)	1.1 (0.68–1.79)	1.04 (0.62–1.75)
Spindle cell melanoma, not otherwise specified	0.404 (0.091–1.802)	0.4 (0.088–1.811)	0.444 (0.1–1.95)	0.44 (0.09–1.96)
Mucosal lentiginous melanoma	0.529 (0.158–1.772)	0.577 (0.171–1.951)	0.59 (0.17–2)	0.62 (0.18–2.15)
Other	0.364 (0.106–1.248)	0.443 (0.129–1.523)	0.26 (0.07–0.93)	0.31 (0.09–1.12)
				
**Tumor stage**				
Localized	1.00 (reference)	1.00 (reference)	1.00 (reference)	1.00 (reference)
Regional by direct extension only	1.1 (0.577–2.097)	1.232 (0.623–2.437)	1.23 (0.64–2.36)	1.37 (0.69–2.73)
Regional lymph nodes involved only	1.664 (1.051–2.635) **	1.86 (1.139–3.037) **	1.78 (1.11–2.84) **	2.02 (1.23–3.33) ***
Regional by both direct extension and lymph node involvement	1.755 (1.047–2.943) **	2.023 (1.177–3.479) **	1.95 (1.13–3.33) **	2.37 (1.35–4.15) ***
Distant	4.491 (3.115–6.476) ***	5.091 (3.424–7.568) ***	4.67 (3.2–6.77) ***	5.31 (3.54–7.98) ***
				
**Living area**				
Counties in metropolitan areas of 1 million persons	1.00 (reference)	1.00 (reference)	1.00 (reference)	1.00 (reference)
Counties in metropolitan areas of 250,000 to 1 million persons	1.114 (0.762–1.629)	1.028 (0.685–1.541)	1.14 (0.78–1.68)	1.05 (0.69–2.73)
Counties in metropolitan areas of 250,000 persons	0.652 (0.351–1.209)	0.668 (0.353–1.263)	0.62 (0.33–1.18)	0.64 (0.33–1.24)
Nonmetropolitan counties adjacent to a metropolitan area	1.28 (0.67–2.446)	1.126 (0.555–2.284)	1.29 (0.67–2.49)	1.14 (0.55–2.36)
Nonmetropolitan counties not adjacent to a metropolitan area	1.067 (0.504–2.259)	1.069 (0.494–2.315)	1.17 (0.53–2.56)	1.16 (0.52–2.61)
				
**Income per year**				
<USD 35,000	1.00 (reference)	1.00 (reference)	1.00 (reference)	1.00 (reference)
USD 35,000–44,999	1.248 (0.414–3.761)	1.639 (0.493–5.445)	1.27 (0.41–3.89)	1.69 (0.49–5.73)
USD 45,000–54,999	0.777 (0.272–2.216)	0.932 (0.296–2.942)	0.8 (0.28–2.31)	0.96 (0.30–3.07)
USD 55,000–64,999	0.558 (0.188–1.66)	0.664 (0.2–2.2)	0.57 (0.19–1.72)	0.67 (0.2–2.28)
USD 65,000–74,999	0.597 (0.197–1.81)	0.74 (0.219–2.5)	0.62 (0.2–1.89)	0.77 (0.22–2.62)
USD 75,000+	0.465 (0.153–1.416)	0.551 (0.162–1.872)	0.49 (0.16–1.49)	0.59 (0.17–2.07)
				
**Marital Status**				
Married	1.00 (reference)	1.00 (reference)	1.00 (reference)	1.00 (reference)
Single	1.116 (0.732–1.703)	1.114 (0.717–1.731)	1.2 (0.79–1.87)	1.23 (0.79–1.93)
Divorced/separated	0.928 (0.539–1.598)	0.658 (0.35–1.24)	0.93 (0.54–1.63)	0.68 (0.36–1.3)
Widowed	1.047 (0.718–1.527)	1.024 (0.69–1.52)	1.01 (0.68–1.48)	1.01 (0.68–1.52)
				
**Surgery and Radiation**				
No	1.00 (reference)	1.00 (reference)	1.00 (reference)	1.00 (reference)
Yes	0.943 (0.645–1.38)	0.939 (0.628–1.402)	0.96 (0.66–1.41)	0.96 (0.64–1.43)
				
**Chemotherapy**				
No	1.00 (reference)	1.00 (reference)	1.00 (reference)	1.00 (reference)
Yes	0.908 (0.645–1.277)	0.852 (0.596–1.217)	0.905 (0.63–1.29)	0.86 (0.59–1.25)

*** *p* < 0.01, ** *p* < 0.05.

## Data Availability

The data used and/or analyzed in this study are available in the Surveillance, Epidemiology, and End Results (SEER) Database of the National Cancer Institute (http://seer.cancer.gov, accessed on 4 April 2020).

## References

[1] American Cancer Society (2020). American Cancer SocietyAtlanta.

[2] Patel J.K., Didolkar M.S., Pickren J.W., Moore R.H. (1978). Metastatic pattern of malignant melanoma. Am. J. Surg..

[3] Augustyn A., de Leon E.D., Yopp A.C. (2015). Primary gastric melanoma: Case report of a rare malignancy. Rare Tumors..

[4] Spiridakis K.G., Polichronaki E.E., Sfakianakis E.E., Flamourakis M.E., Margetousakis T.H., Xekalou A.S., Lianeris G., Giannikaki E., Christodoulakis M. (2015). Primary small bowel melanoma. A case report and a review of the literature. G. Di Chir..

[5] Ait Idir B., Riany A., Jahid A., Chad B. (2016). Primary melanoma of the small bowel revealed by gastrointestinal bleeding: A case report. J. Med. Case Rep..

[6] Simons M., Ferreira J., Meunier R., Moss S. (2016). Primary versus Metastatic Gastrointestinal Melanoma: A Rare Case and Review of Current Literature. Case Rep. Gastrointest. Med..

[7] Timmers T.K., Schadd E.M., Monkelbaan J.F., Meij V. (2013). Survival after Resection of a Primary Malignant Melanoma of the Small Intestine in a Young Patient: Report of a Case. Case Rep. Gastroenterol..

[8] Chu Y.-M., Hung C.-S., Huang C.-S. (2021). Primary malignant melanoma of the esophagogastric junction: A case report. Medicine.

[9] Yamaguchi T., Fushida S., Kinoshita J., Saito H., Shimada M., Terai S., Moriyama H., Okamoto K., Nakamura K., Ninomiya I. (2021). A case of primary malignant melanoma of the esophagogastric junction with abscopal effect after nivolumab administration. Surg. Case Rep..

[10] Akaraviputh T., Trakarnsanga A., Murph P.M. (2011). Surgical Management of Malignant Melanoma of Gastrointestinal Tract. Melanoma in the Clinic—Diagnosis, Management and Complications of Malignancy.

[11] Zheng Y., Cong C., Su C., Sun Y., Xing L. (2020). Epidemiology and survival outcomes of primary gastrointestinal melanoma: A SEER-based population study. Int. J. Clin. Oncol..

[12] Duggan M.A., Anderson W.F., Altekruse S., Penberthy L., Sherman M.E. (2016). The Surveillance, Epidemiology, and End Results (SEER) Program and Pathology: Toward Strengthening the Critical Relationship. Am. J. Surg. Pathol..

[13] Kottschade L.A., Grotz T.E., Dronca R.S., Salomao D.R., Pulido J.S., Wasif N., Jakub J.W., Bagaria S.P., Kumar R., Kaur J.S. (2014). Rare Presentations of Primary Melanoma and Special Populations: A Systematic Review. Am. J. Clin. Oncol..

[14] Al-Haseni A., Vrable A., Qureshi M.M., Mathews S., Pollock S., Truong M.-T., Sahni D. (2019). Survival outcomes of mucosal melanoma in the USA. Future Oncol..

[15] Lian B., Cui C.L., Zhou L., Song X., Zhang X.S., Wu D., Si L., Chi Z., Sheng X., Mao L. (2017). The natural history and patterns of metastases from mucosal melanoma: An analysis of 706 prospectively-followed patients. Ann. Oncol..

[16] Raycraft T., Cheung W.Y., Yin Y., Speers C., Ko J.J., Mariano C. (2019). Causes of mortality in older patients with stage 3 colon cancer. J. Geriatr. Oncol..

[17] Siegel R.L., Miller K.D., Fuchs H.E., Jemal A. (2022). Cancer statistics, 2022. CA Cancer J. Clin..

[18] Khalid U., Saleem T., Imam A.M., Khan M.R. (2011). Pathogenesis, diagnosis and management of primary melanoma of the colon. World J. Surg. Oncol..

[19] Hadjinicolaou A.V., Hadjittofi C., Athanasopoulos P.G., Shah R., Ala A.A. (2016). Primary small bowel melanomas: Fact or myth?. Ann. Transl. Med..

[20] Tang L., Pan Z., Zhang X. (2022). The effect of marital status on the survival of patients with multiple myeloma. Hematology.

[21] Wang S., Chen L., Chen D., Chao J., Shao Y., Tang K., Chen W. (2021). Effect of Marital Status on the Survival of Patients With Adenocarcinoma of the Esophagogastric Junction: A Population-Based, Propensity-Matched Study. Cancer Control..

[22] Zhou C., Zhang Y., Hu X., Fang M., Xiao S. (2021). The effect of marital and insurance status on the survival of elderly patients with stage M1b colon cancer: A SEER-based study. BMC Cancer.

[23] Alyabsi M., Ramadan M., Algarni M., Alshammari K., Jazieh A.R. (2021). The effect of marital status on stage at diagnosis and survival in Saudis diagnosed with colorectal cancer: Cancer registry analysis. Sci. Rep..

[24] Xiao K., Zhao Y., Cai Y., Chen P., Chen J., Ye R., Liu X., Yuan B., Zhao Y. (2020). The effect of marital status on the survival of patients with colorectal neuroendocrine neoplasms: An analysis of the SEER database. Rev. Esp. Enferm. Dig..

[25] Dong J., Dai Q., Zhang F. (2019). The effect of marital status on endometrial cancer-related diagnosis and prognosis: A Surveillance Epidemiology and End Results database analysis. Future Oncol..

[26] Hinyard L., Wirth L.S., Clancy J.M., Schwartz T. (2017). The effect of marital status on breast cancer-related outcomes in women under 65: A SEER database analysis. Breast.

[27] Xie J.C., Yang S., Liu X.Y., Zhao Y.X. (2018). Effect of marital status on survival in glioblastoma multiforme by demographics, education, economic factors, and insurance status. Cancer Med..

[28] Liang Y., Wu X., Lu C., Xiao F. (2021). Impact of marital status on the prognosis of liver cancer patients without surgery and the critical window. Ann. Palliat. Med..

[29] Feng Y., Dai W., Li Y., Mo S., Li Q., Cai S. (2018). The effect of marital status by age on patients with colorectal cancer over the past decades: A SEER-based analysis. Int. J. Colorectal Dis..

